# Second-line immunosuppressant administration for steroid-refractory immune-related adverse events in patients with lung cancer

**DOI:** 10.1007/s00262-023-03528-x

**Published:** 2023-08-28

**Authors:** Shinsuke Ogusu, Yuhei Harutani, Takehiro Tozuka, Ryota Saito, Junji Koyama, Hiroaki Sakamoto, Tomoaki Sonoda, Yuko Tsuchiya-Kawano, Tomohiro Oba, Keita Kudo, Hiroshi Gyotoku, Katsumi Nakatomi, Ryo Ariyasu

**Affiliations:** 1 Division of Hematology, Respiratory Medicine and Oncology, Department of Internal Medicine, Faculty of Medicine, Saga University, Saga, Japan; 2 Internal Medicine III, Wakayama Medical University, Wakayama, Japan; 3 Department of Pulmonary Medicine and Oncology, Graduate School of Medicine, Nippon Medical School, Tokyo, Japan; 4 Department of Respiratory Medicine, Tohoku University Graduate School of Medicine, Sendai, Japan; 5 Department of Respiratory Medicine, Nagoya University Graduate School of Medicine, Nagoya, Japan; 6 Department of Respiratory Medicine, Hirosaki University Graduate School of Medicine, Hirosaki, Japan; 7 Division of Respiratory Medicine, University of Fukui Hospital, Yoshida-Gun, Fukui Japan; 8 Department of Respiratory Medicine, Kitakyushu Municipal Medical Center, Kitakyushu, Japan; 9 Department of Respiratory Medicine, Saitama Red Cross Hospital, Saitama, Japan; 10 Department of Medical Oncology, NHO Osaka Minami Medical Center, Kawachinagano-shi, Osaka Japan; 11 Department of Respiratory Medicine, Nagasaki University Graduate School of Biomedical Sciences, Nagasaki University, Nagasaki, Japan; 12 Department of Respiratory Medicine, National Hospital Organization Ureshino Medical Center, Ureshino-shi, Saga Japan; 13 Department of Thoracic Medical Oncology, The Cancer Institute Hospital, Japanese Foundation for Cancer Research, 3-8-31, Ariake, Koto-ku, Tokyo 135-8550 Japan

**Keywords:** Second-line immunosuppressants, Immune-related adverse events, Corticosteroid-refractory, Lung cancer

## Abstract

**Background:**

Evidence for use of second-line immunosuppressants for immune-related adverse events (irAEs) is inadequate. Therefore, a multicenter analysis should assess the efficacy of second-line immunosuppressants for severe irAEs associated with different malignant diseases.

**Methods:**

This descriptive study aims to investigate the effects of second-line immunosuppressants on corticosteroid-refractory irAEs in patients with lung cancer. We analyzed the effects of second-line immunosuppressants on underlying lung cancer and associated adverse effects.

**Results:**

Our study included 4589 patients who had received immune checkpoint inhibitor treatment, with 73 patients (1.6%) developing irAEs requiring second-line immunosuppressants. The most commonly observed irAE was pneumonitis (26 patients), followed by hepatobiliary disorders (15 patients) and enteritis (14 patients). We found a confirmed response rate of 42.3% for pneumonitis, which was lower than the response rates of 86.7% for hepatobiliary disorders and 92.9% for enteritis. The time from the start of corticosteroid therapy to the addition of a second-line immunosuppressant correlated significantly with the resolution of irAE to Grade 1 (correlation coefficients of r = 0.701, *p* < 0.005). The median progression-free survival and duration of response of underlying lung cancer from second-line immunosuppressant administration were 2.1 and 3.0 months, respectively. Of the patients with irAE, 27.4% developed infections and 5.5% might die due to infection.

**Conclusion:**

Second-line immunosuppressant response was confirmed in 72.2% of irAEs in patients with lung cancer, with lower response rates observed in irAE pneumonitis compared to other irAEs.

**Supplementary Information:**

The online version contains supplementary material available at 10.1007/s00262-023-03528-x.

## Introduction

Immune checkpoint inhibitors (ICIs), including anti-PD-1/PD-L1 and anti-CTLA-4 antibodies, have become the standard of care for lung cancer. ICIs are used for systemic therapy in advanced disease [1–3], consolidation therapy after chemoradiation therapy [4], neo-adjuvant therapy [5], and adjuvant therapy [6]. However, during and after ICI treatment, immune-related adverse events (irAEs) can occur, which are not typically seen with conventional chemotherapy and molecular target therapy [7]. IrAEs are caused by an excessive immune response not only to tumor cells but also to normal tissues, leading to organ problems and sometimes even death although severe irAEs are rare. Therefore, accurately managing irAEs is crucial in current lung cancer practice.

For moderate to severe irAEs, corticosteroids are typically used as a first-line treatment. In cases of severe irAEs not controlled by corticosteroids alone, the addition of second-line immunosuppressants, such as infliximab or mycophenolate mofetil, may be considered. These second-line immunosuppressants are expected to increase immunosuppression and resolve severe irAEs. However, evidence supporting the use of second-line immunosuppressants for irAE management has been insufficient despite clinical guideline recommendations [8, 9]. Most previous reports on second-line immunosuppressants for irAE management are single-institution retrospective analyses [10, 11], which are subject to treatment bias. Additionally, in most reports, second-line immunosuppressants were primarily used for melanoma patients although differences in the clinical course are predicted between primary malignant diseases [12, 13]. Therefore, conducting a multicenter analysis of second-line immunosuppressants for severe irAEs in patients with lung cancer would be important to establish robust evidence for their use in irAE management.

In this study, we conducted a multicenter retrospective analysis of second-line immunosuppressants for irAEs in patients with lung cancer. We analyzed the effect of second-line immunosuppressants and their adverse effect on the underlying disease.

## Materials and methods

### Study design and patient population

We conducted a multicenter retrospective analysis in the TOPGAN group, a Japanese lung cancer research group (TOPGAN 2022-01), with the primary objective of clarifying the effect of second-line immunosuppressants for corticosteroid-refractory irAEs. We defined steroid refractoriness as irAEs that were not controlled by steroids alone. In such cases, second-line immunosuppressants were also administered. Second-line immunosuppressants were also used to taper steroid after irAEs were under control. We collected data on patients with lung cancer who had received ICI treatment until July 2022 and had developed irAEs and received second-line immunosuppressants in addition to corticosteroids. Patient data collected included patient characteristics (age, sex, etc.), type of irAE, effect of second-line immunosuppressants, and clinical course after second-line immunosuppressant administration. Second-line immunosuppressants analyzed in this study were azathioprine, cyclophosphamide, cyclosporine, infliximab, intravenous immunoglobulin, mycophenolate mofetil, tacrolimus, and tocilizumab. This study was approved by the institutional review board of the Cancer Institute Hospital of the Japanese Foundation for Cancer Research (IRB no. 2022-GB-065), and informed consent was waived because of the retrospective nature of the study, with an opt-out option included.

Two outcomes were evaluated in this study: (1) Responses to second-line immunosuppressants determined by each investigator using patient medical records and (2) The resolution of irAEs to CTCAE grade 1 (G1) within 90 days of second-line immunosuppressant initiation. The clinical course of the underlying lung cancer was evaluated by measuring progression-free survival (PFS) and duration of response (DOR) from the initiation of second-line immunosuppressant administration. Tumor response was evaluated based on the RECIST v1.1 criteria. Incidence of infections following second-line immunosuppressant administration was also analyzed.

### Statistical analysis

Categorical variables were compared using Fisher’s exact test, and continuous data were compared using the Mann–Whitney U test. The strength of the relationship between two sets of data was determined using Spearman’s rank correlation coefficient. The Kaplan–Meier method was used to estimate the median survival time. Statistical significance was set at *p* < 0.05. Data analysis was performed using R and SPSS Statistics for Windows version 24.

## Results

### Patient characteristics

**Fig. 1:**
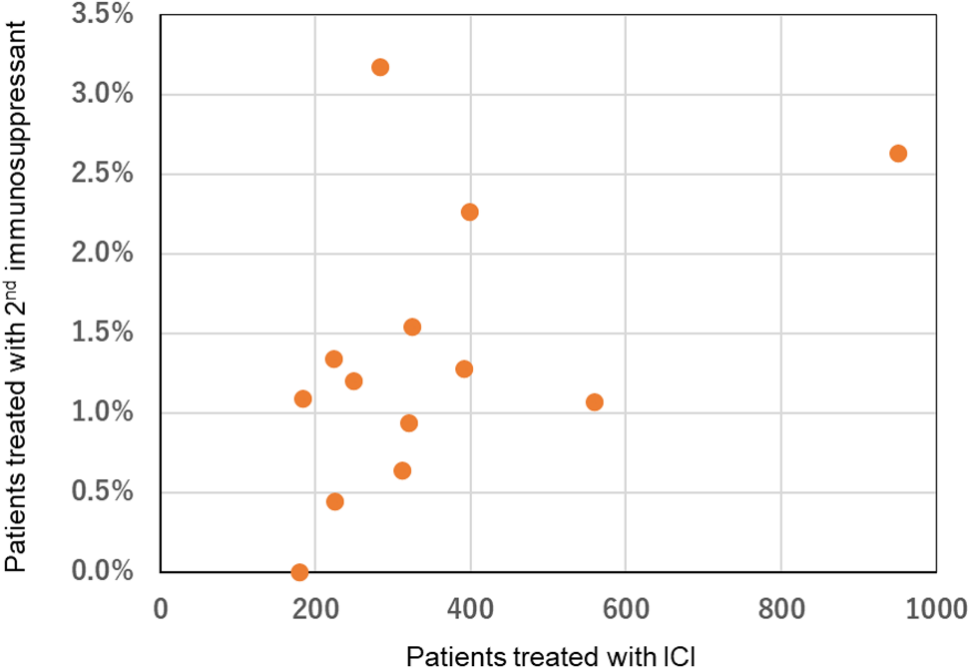
The frequency of patients requiring second-line immunosuppressants in each institution

Overall, 73 of 4589 patients who received ICI treatment (1.6%) developed irAEs requiring second-line immunosuppressant administration. Of the 358 patients who received combination immunotherapy with nivolumab and ipilimumab, 17 (4.7%) developed irAEs requiring second-line immunosuppressants. The frequency of patients requiring second-line immunosuppressants varied by institution, ranging from 0 to 3.2% (Fig. 1).

The characteristics of the 73 patients who required second-line immunosuppressants are summarized in Table 1. Among them, 15 patients (20.5%) were female, 70 (95.9%) had non–small cell lung cancer, 5 (6.8%) had a history of autoimmune disease (3 with rheumatoid arthritis, 1 with dermatomyositis, and 1 with scleroderma), and 18 (24.7%) had a history of thoracic radiation therapy. Most patients (71.2%) developed grade ≥ 3 irAEs when second-line immunosuppressants were administered, and 64 patients (87.7%) were treated with corticosteroids (prednisolone ≥ 1 mg/kg).

**Table 1:** Patient characteristics

N	73
Age < 75 >, 75 <)	58/15
Sex (F/M)	15/58
Smoking history (current or past/never	65/8
Performance Status (0,1/2)	67/6
Histology (NSCLC/SCLC)	70/3
PD-L1 status (positive/negative/unknown)	40/12/21
Baseline prednisolone usage (10 mg >, 10 mg <)	70/3
History of autoimmune disease (positive/negative)	5/68
History of thoracic radiation (positive/negative)	18/55
Treatment line (1st line, consolidation/2nd or later line)	50/23
ICI/Chemo + ICI/ICI combination/experimental	36/17/17/3
ICI response (CR/PR/SD/PD/NE)	3/33/19/7/11
irAE grade (1/2/3/4)	5/16/39/13
Maximum steroid usage for irAE (lmg/kg >/< /unknown)	4/64/5

*CR* complete response, *PR* partial response, *SD* stable disease, *PD* progressive disease, *NE* not evaluable

### Response of second-line immunosuppressants for irAE

**Fig. 2:**
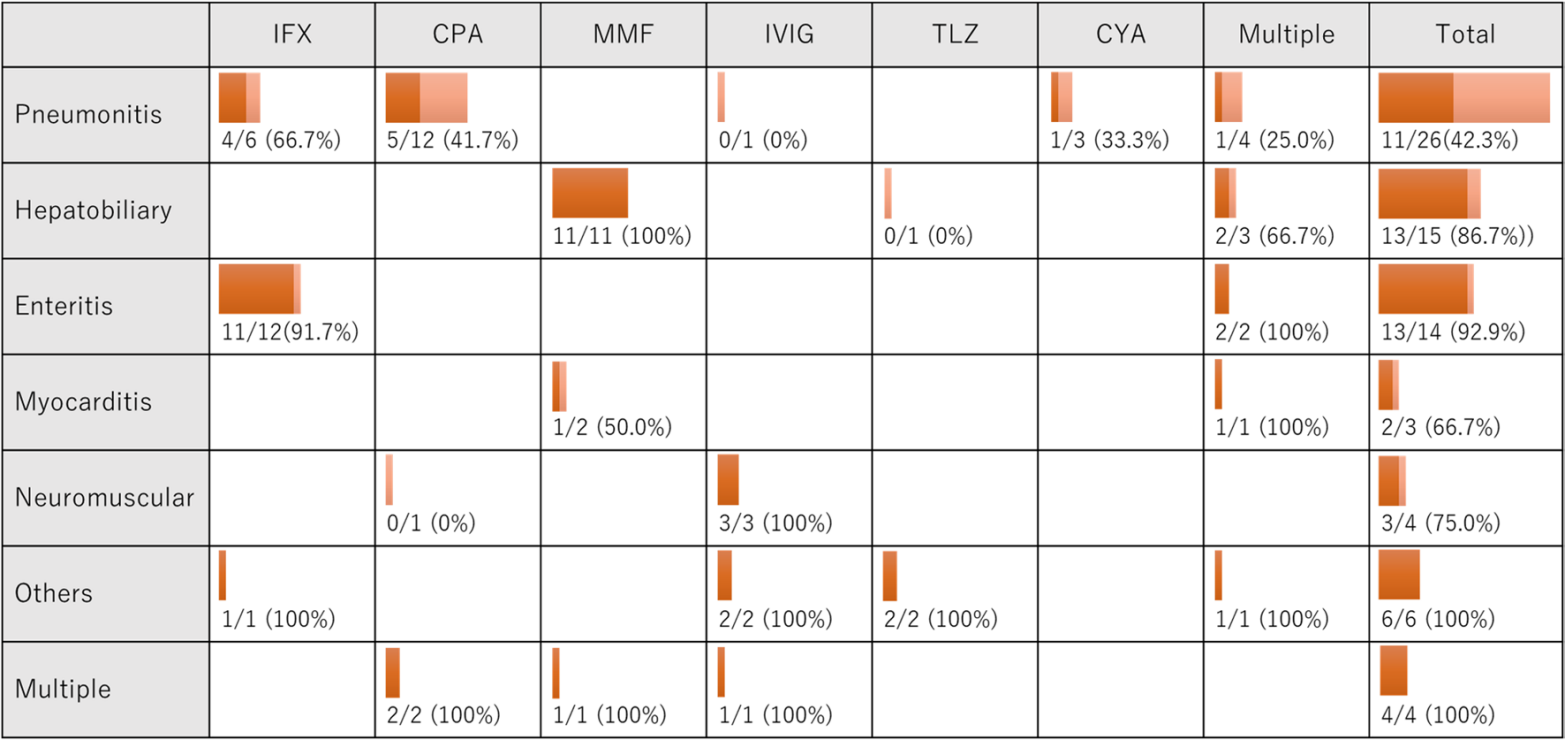
Response rates of second-line immunosuppressants in each irAE. Abbreviations: *IFX* infliximab, *CPA* cyclophosphamide, *MMF* mycophenolate mofetil, *IVIG* intravenous immunoglobulin, *TLZ* tocilizumab, *CYA* cyclosporine. Multiple = treated with multiple second-line immunosuppressants

Response of second-line immunosuppressants was evaluated in 72 of 73 cases (in one case, it was unclear whether respiratory failure was due to irAE or lung cancer progression). The most common irAE was pneumonitis (n = 26), followed by hepatobiliary disorders (n = 15) and enteritis (n = 14). Infliximab was the most frequently used immunosuppressant (n = 19), followed by cyclophosphamide (n = 15) and mycophenolate mofetil (n = 14). Of the 72 cases evaluated, 52 patients (72.2%) responded to the second-line immunosuppressants. Response rates of second-line immunosuppressants in each irAE are shown in Fig. 2. Response was confirmed in 42.3% of pneumonitis cases (11/26), which was lower than the response rates of 86.7% in hepatobiliary disorders (13/15) and 92.9% in enteritis (13/14). Response based on whether the irAEs resolved to G1 or not were shown in the Supplemental material.

### Pneumonitis analysis

Among the patients with pneumonitis, 34.6% (9/26) had a history of thoracic irradiation (with definitive radiotherapy performed in all nine cases). The median time between radiation completion and pneumonitis onset was 95 (range, 31–784) days. Responsiveness to second-line immunosuppressants was confirmed in 44.4% (4/9) of cases. This was not substantially different from the 41.2% (7/17) response rate seen in those who had not undergone irradiation. The types of pneumonitis and number of cases among our patients were organizing pneumonia (OP) in 11 cases, nonspecific interstitial pneumonia (NSIP) in five cases, hypersensitivity pneumonia (HP) in one case, diffuse alveolar damage (DAD) in eight cases, and OP plus DAD in one case. The response rates to second-line immunosuppressants for each type were OP, 63.6% (7/11); NSIP, 60.0% (3/5); HP, 100% (1/1); DAD, 0% (0/8); and OP + DAD, 0% (0/1).

### Correlation of timing and response of second-line immunosuppressants

**Fig. 3:**
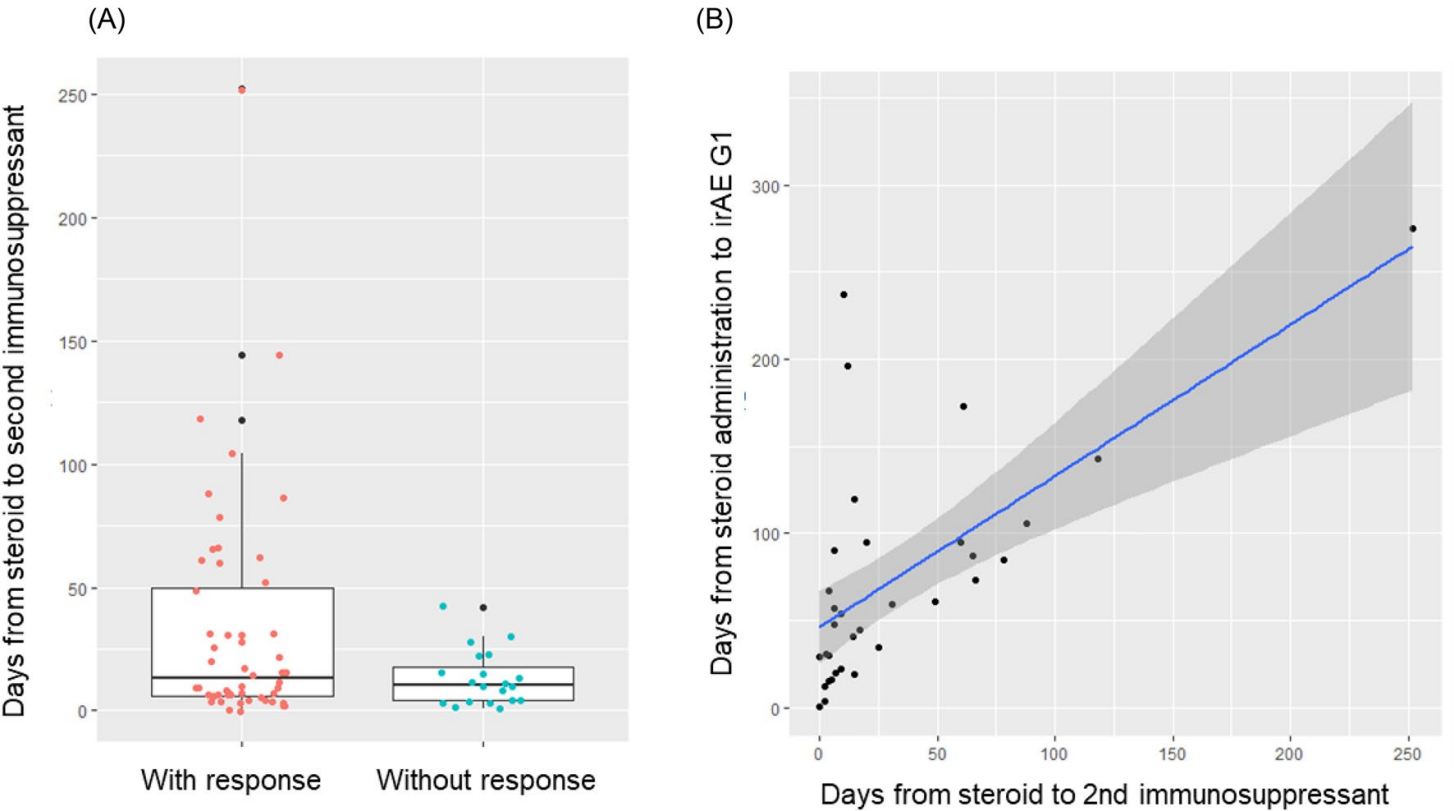
**A** The median time from the start of corticosteroid administration to second-line immunosuppressant addition in responder and non-responder. **B** The correlation between the time from the start of corticosteroid administration to immunosuppressant addition and irAE resolution to G1

In all 72 patients evaluated, the median time from the start of corticosteroid administration to second-line immunosuppressant addition was 13.0 days in patients with a response and 10.5 days in patients without a response (*p* = 0.148). In the 34 patients whose irAE resolved to G1 after second-line immunosuppressant addition, there was a significant correlation between the time from the start of corticosteroid administration to immunosuppressant addition and irAE resolution to G1 (a correlation coefficient of r = 0.701, *p* < 0.005). (Fig. 3).

### Underlying lung cancer and infection after addition of second-line immunosuppressants

**Fig. 4:**
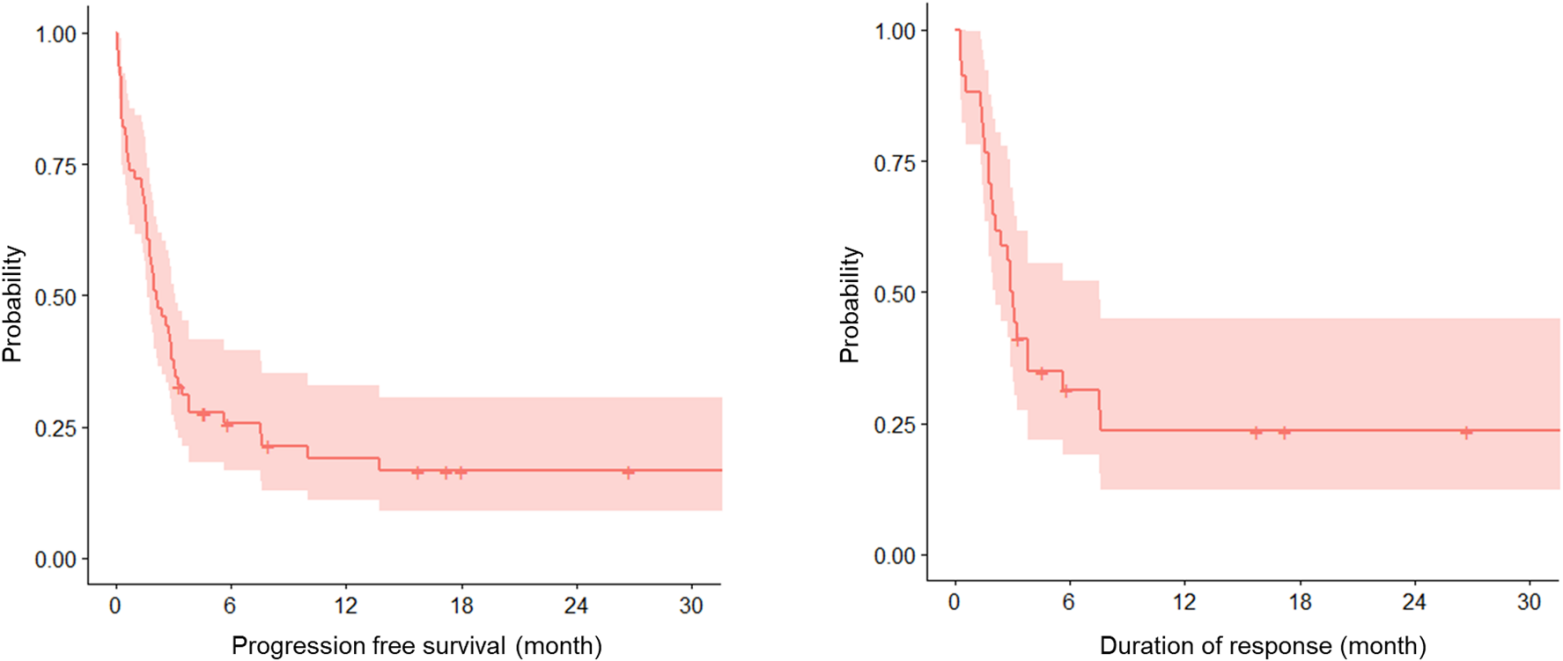
The progression-free survival and duration of response of underlying lung cancer from the initiation of second-line immunosuppressant administration

**Fig. 5:**
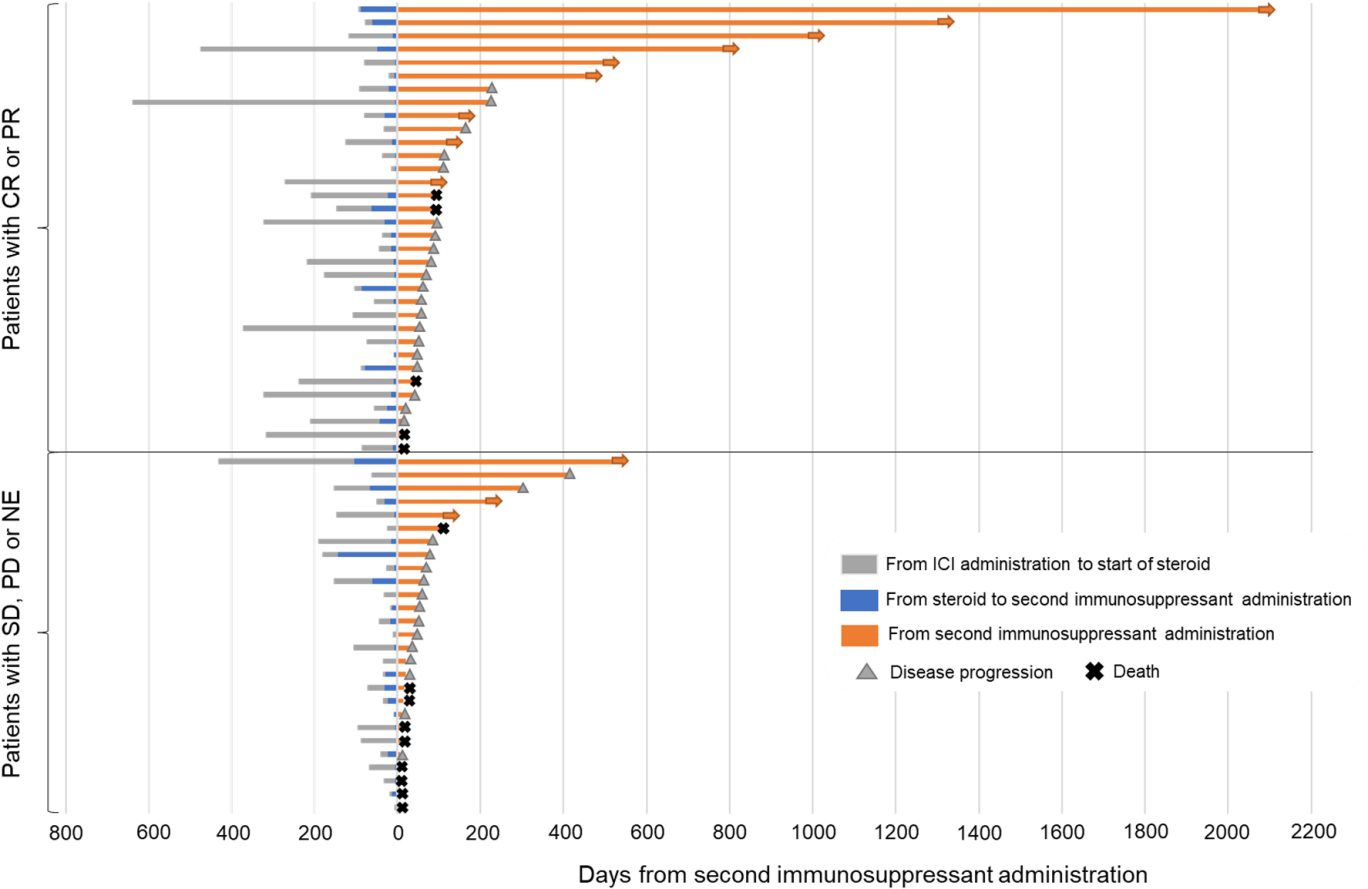
The detailed clinical course of each patient

In the 61 patients who did not experience lung cancer progression in second-line immunosuppressant addition, the median PFS was 2.1 months. Among the 34 patients who remained in partial response of lung cancer in immunosuppressant addition, the median DOR was 3.0 months (Fig. 4). The detailed clinical course of each patient is shown in Fig. 5. Of the 73 patients, 20 (27.4%) developed infections after second-line immunosuppressants were added to their treatment. Ten patients had a cytomegalovirus infection, and ten had pneumonia (two cases each of pneumococcus pneumonia and pulmonary aspergillosis). The median time between the initiation of steroid treatment to the onset of infection was 46 days (range, 3–223). From the initiation of second-line immunosuppressant treatment to the onset of infection was 57 days (range, 17–272). Four patients (5.5%) might die due to infection, on top of their underlying lung cancer after the addition of second-line immunosuppressants to their treatment. The infections in these four cases were pneumonia and urinary tract infection, Legionella pneumonia, cytomegalovirus infection and pneumococcus pneumonia, and cytomegalovirus infection. The time between the initiation of second-line immunosuppressants to the onset of infection was 5, -3, 19, 39 days, respectively (In one case, infection had already occurred at the time of adding the second-line immunosuppressants). Second-line immunosuppressants were administered just once in the former two cases, while in the other two, they were continued for 15 and 42 days.

## Discussion

We collected data on patients with lung cancer who received second-line immunosuppressants for corticosteroid-refractory irAEs. The second-line immunosuppressants were used in 1.6% and 4.7% of patients who received anti-PD-1/PD-L1 and anti-CTLA-4 antibody combination therapy, respectively. The combination of anti-CTLA-4 and anti-PD-1/PD-L1 antibodies increases the rate of irAEs, which may explain the higher rate of second-line immunosuppressant usage in our study [14, 15]. We found that the response rate of second-line immunosuppressants was confirmed in 72.2% of patients, with a lower response rate observed in patients with pneumonitis (42.3%) compared to those with hepatobiliary disorders and enteritis.

A previous single-center report also showed a low response rate (30.0%) of second-line immunosuppressants in irAE pneumonitis in patients with lung cancer [10]. Other reports also demonstrated a similar tendency, where the administration of infliximab showed a response in 30% of severe irAE pneumonitis cases [11, 16]. In our report, the most commonly used second-line immunosuppressant for irAE pneumonitis was cyclophosphamide, which is different from past reports, but the response rate was similarly low. Cyclophosphamide has been reported to be ineffective in the acute exacerbation of idiopathic pulmonary fibrosis [17], and the same trend was confirmed in this study. Thus, the management and selection of second-line immunosuppressants for irAE pneumonitis need improvement compared to other irAEs.

Guidelines for managing irAEs recommend considering the addition of a second-line immunosuppressant 3–4 days after starting corticosteroid therapy [9]. A retrospective study of irAE colitis showed that early administration of a second-line immunosuppressant resulted in shorter hospitalization and symptom duration [18]. The time from the start of corticosteroid therapy to the addition of a second-line immunosuppressant correlated significantly with the time to resolution of irAE to G1 in this study. However, there was no clear correlation between the timing of the second-line immunosuppressant administration and response. Future studies should investigate the timing of second-line immunosuppressant administration, taking into account possible adverse effects, as discussed below.

The previous clinical trials have reported that patients with lung cancer who discontinued treatment due to severe irAEs tend to have a longer DOR than others [19]. However, a retrospective study showed that patients with melanoma who received second-line immunosuppressants tended to have a shorter overall survival time than others [20]. In this report, the PFS and DOR of lung cancer after second-line immunosuppressant administration were comparatively short (2.1 and 3.0 months, respectively). This indicates that excessive immunosuppression could lead to the progression of lung cancer as anti-TNF treatment has been reported to increase the risk of cancer [21]. However, no comparison has been made with a single arm, so it is impossible to draw any definitive conclusions. Moreover, in this report, 27.4% of patients developed an infection, and 5.5% of patients might die due to an infection. The adverse effects of second-line immunosuppressants may not be negligible in clinical practice.

The evidence supporting the recommendation of second-line immunosuppressants has been relatively insufficient, relying mainly on retrospective studies from single centers and expert opinions. As shown in this multicenter study, irAE management with second-line immunosuppressants is not always sufficient to resolve all irAEs, particularly pneumonitis, and adverse effects on underlying lung cancer are a concern. Therefore, it is crucial to accumulate further evidence on the use of second-line immunosuppressants in each malignant disease, including this study.

There are some limitations to this study. First, as this is a case series, we were unable to compare second-line immunosuppressants with other treatments. Second, due to the retrospective nature of this study, some biases in treatment selection and effects could not be avoided despite conducting multicenter trials to minimize these biases.

## Conclusion

The response rate of second-line immunosuppressants was confirmed in 72.2% of irAEs in patients with lung cancer. However, the response rate was lower in irAE pneumonitis compared to other irAEs.

### Supplementary Information

Below is the link to the electronic supplementary material.

Supplementary file1 (DOCX 62 KB)

## Data Availability

The datasets generated and/or analyzed during the current study are available from the corresponding author on reasonable request.

## Abbreviations

DOR: Duration of response
G1: Grade 1
ICI: Immune checkpoint inhibitor
irAEs: Immune-related adverse events
PFS: Progression-free survival
