# Supplementary material for: Second-line immunosuppressant administration for steroid-refractory immune-related adverse events in patients with lung cancer
Source: Cancer Immunol Immunother. 2023 Aug 28;72(11):3765–72. doi: 10.1007/s00262-023-03528-x (PMC10576678; doi:10.1007/s00262-023-03528-x)

*Response of second-line immunosuppressants for irAE*

In 52 cases, it was possible to evaluate whether irAEs resolved to G1 within 90 days from the administration of second-line immunosuppressants. Among them, pneumonitis resolved to G1 in 20.0% (4/20), which was lower than the resolution rates of 66.7% in hepatobiliary disorders (6/9) and 90.9% in enteritis (10/11).


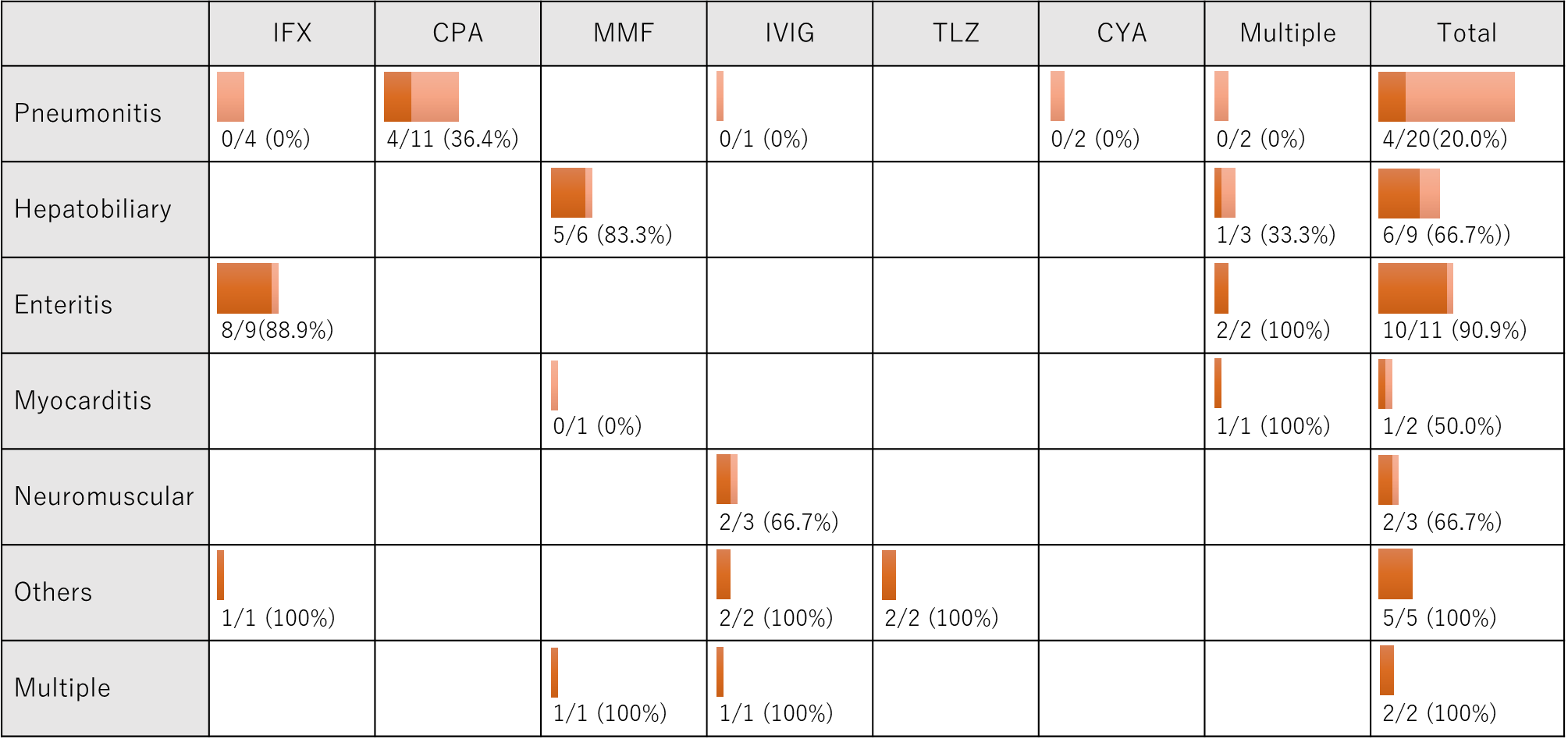

Supplement: Supplementary file 1 — Supplementary file1 (DOCX 62 KB) [file 262_2023_3528_MOESM1_ESM.docx]
